# Purkinje Cell Activity in the Medial and Lateral Cerebellum During Suppression of Voluntary Eye Movements in Rhesus Macaques

**DOI:** 10.3389/fncel.2022.863181

**Published:** 2022-04-28

**Authors:** Eric Avila, Nico A. Flierman, Peter J. Holland, Pieter R. Roelfsema, Maarten A. Frens, Aleksandra Badura, Chris I. De Zeeuw

**Affiliations:** ^1^Netherlands Institute for Neuroscience, Amsterdam, Netherlands; ^2^Department of Neuroscience, Erasmus MC, Rotterdam, Netherlands; ^3^School of Psychology, University of Birmingham, Birmingham, United Kingdom; ^4^Department of Integrative Neurophysiology, VU University, Amsterdam, Netherlands; ^5^Department of Psychiatry, Academic Medical Centre, Amsterdam, Netherlands

**Keywords:** cerebellum, simple spikes, executive control, antisaccades, flexible behavior, non-human primate (NHP)

## Abstract

Volitional suppression of responses to distracting external stimuli enables us to achieve our goals. This volitional inhibition of a specific behavior is supposed to be mainly mediated by the cerebral cortex. However, recent evidence supports the involvement of the cerebellum in this process. It is currently not known whether different parts of the cerebellar cortex play differential or synergistic roles in the planning and execution of this behavior. Here, we measured Purkinje cell (PC) responses in the medial and lateral cerebellum in two rhesus macaques during pro- and anti-saccade tasks. During an antisaccade trial, non-human primates (NHPs) were instructed to make a saccadic eye movement away from a target, rather than toward it, as in prosaccade trials. Our data show that the cerebellum plays an important role not only during the execution of the saccades but also during the volitional inhibition of eye movements toward the target. Simple spike (SS) modulation during the instruction and execution periods of pro- and anti-saccades was prominent in PCs of both the medial and lateral cerebellum. However, only the SS activity in the lateral cerebellar cortex contained information about stimulus identity and showed a strong reciprocal interaction with complex spikes (CSs). Moreover, the SS activity of different PC groups modulated bidirectionally in both of regions, but the PCs that showed facilitating and suppressive activity were predominantly associated with instruction and execution, respectively. These findings show that different cerebellar regions and PC groups contribute to goal-directed behavior and volitional inhibition, but with different propensities, highlighting the rich repertoire of the cerebellar control in executive functions.

## Introduction

In a dynamic environment, volitional control of behavior is necessary to make flexible, well-adapted choices. This often requires selective suppression of responses to external stimuli, a hallmark of conscious, executive control (Diamond, 2013). In a laboratory setting, we can measure this complex behavior using the antisaccade task which requires the subjects to refrain from looking at a suddenly appearing target and instead execute a saccade to the (unmarked) mirror position of that target (Everling and Fischer, 1998; Mitchell et al., 2002; Munoz and Everling, 2004). Similar to other volitional movements, many regions in the cerebral cortex have been identified as crucial for the correct execution of the antisaccade task (Funahashi et al., 1993; Schlag-Rey et al., 1997; Gottlieb and Goldberg, 1999; Bunge et al., 2005; Hakvoort Schwerdtfeger et al., 2012; Everling and Johnston, 2013; Cutsuridis et al., 2014). Given its reciprocal connections with the relevant neocortical regions (Kelly and Strick, 2003), the cerebellum may form an additional hub in this voluntary motor control circuitry (Tanaka et al., 2003; Peterburs et al., 2012; Brunamonti et al., 2014; Kunimatsu et al., 2016; Dacre et al., 2021). This possibility is supported by recent findings that the cerebellum participates in movement planning (Ashmore and Sommer, 2013; Giovannucci et al., 2017; Deverett et al., 2018; Gao et al., 2018; Kostadinov et al., 2019). However, to what extent different parts of the cerebellum contribute to the planning and execution of antisaccades, and complex movements in general, is unclear (Miall et al., 1993; Thach, 2007; Ito, 2008; Gao et al., 2018; Chabrol et al., 2019; De Schutter, 2019).

Several studies have indicated that the execution of simple or reflexive movements may be controlled by Purkinje cells (PCs) in the medial part of the cerebellum, whereas complex behaviors, which require instruction, inhibition, and planning, might be controlled by more lateral regions (Strick et al., 2009; Caligiore et al., 2019; Chabrol et al., 2019; De Schutter, 2019; Sendhilnathan et al., 2020). Other studies, however, advocate that both the simple and complex movements can be controlled by the same group of PCs (Gao et al., 2018, 2019), the location of which in the cerebellar cortex may be determined by the PC-to-effector pathway (Voogd et al., 2012; De Zeeuw, 2021). Settling these questions would require monitoring the spiking activity of PCs in both the medial and the lateral cerebellar areas during a motor task that includes the execution of both simple and complex forms of movements related to the same effector.

**FIGURE 1:**
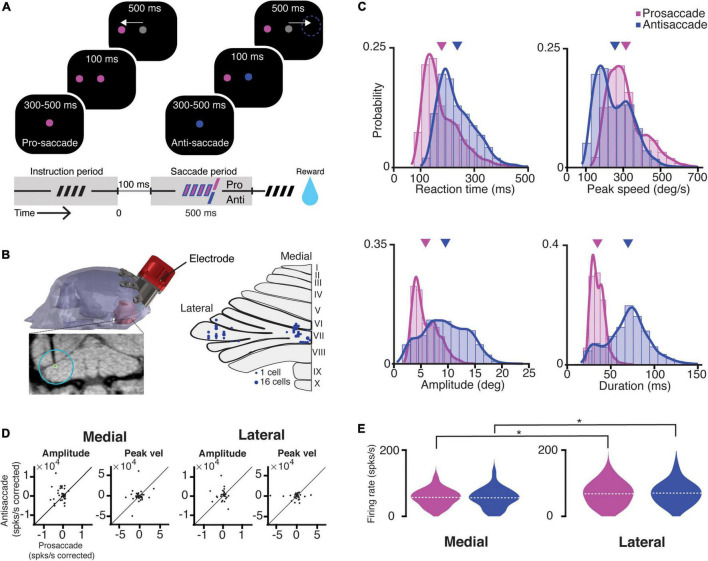
Task, recording location, saccade kinematics, and related PC firing characteristics. **(A)** A scheme representing the different periods of the task. The trial condition was indicated by the color of the fixation point. After a random delay between 300 and 500 ms (i.e., instruction period, dashed symbol), a target appeared in one of the eight locations, while NHPs were required to maintain the fixation for 100 ms. Next, the fixation target switched to gray, and the NHPs were given 500 ms to execute a saccade (i.e., saccade period) toward the target (prosaccade) or the mirror position of the target (antisaccade). **(B)** Left, MRI-based 3D-reconstruction of the skull of Mi; bottom, coronal MRI section showing the localization of an electrode in the left lateral cerebellum; right, *an* overview of the localization of recording sites in the medial (vermal lobules VIc and VII) and lateral cerebellum (crus-I/II, left hemisphere). **(C)** Features of kinematics of all prosaccades (magenta; monkey Mo *n* = 6114, monkey Mi *n* = 6670) and antisaccades (blue; Mo *n* = 4114, Mi *n* = 6305) for all trials over the 122 recording sessions. Antisaccades were characterized by longer reaction times (pro- mean 178.4 ms ± 70.6 SD; anti- mean 236.6 ms ± 72.7 SD; *p* < 0.0001), lower peak velocities (pro- mean 311.8°/s ± 102.6 SD; anti- mean 253.4°/s ± 103.1 SD; *p* < 0.0001), bigger amplitudes (pro- mean 5.8° ± 2.3 SD; anti- mean 9.5° ± 4.1 SD; *p* < 0.0001), and longer durations (pro- mean 34.7 ms ± 7.9 SD; anti- mean 69.8 ms ± 24 SD; *p* < 0.0001). Triangles in magenta and purple indicate means of pro- and anti-saccade movement parameters, respectively. **(D)** Scatter plots with corrected PC activity after a linear regression model fitted to an amplitude or a peak speed during all pro- and antisaccades in the medial (left) and lateral (right) cerebellum. **(E)** Violin plots showing the mean average simple spike firing rates for all the cells during prosaccades (magenta) and antisaccades (blue) for the medial (left) and lateral (right) cerebellum). Dashed lines depict the mean value of the population. The asterisks show significance levels for a Wilcoxon rank-sum test for the comparison between the medial and lateral cerebellum in each condition (pro medial vs. lateral *p* = 0.04; anti medial vs. lateral *p* = 0.03).

Here, we investigated the hypotheses that (1) PCs in the cerebellum play a role in the volitional inhibition of stimulus-driven eye movements during the antisaccade task; and (2) that PCs in the medial and the lateral cerebellum differentially contribute to pro- and anti-saccades. More specifically, we set out to study the modulation of randomly selected saccade-related PCs in the medial (oculomotor vermis, OMV) and the lateral (crus-I/II) cerebellum of non-human primates (NHPs; Macaca mulatta) during the generation of both pro- and anti-saccades (Figure 1). We found that simple spike (SS) modulation during the instruction and execution periods of pro- and anti-saccade trials was prominent in PCs in both cerebellar regions. However, whereas the SS activity in the lateral cerebellum contained information about the stimulus identity, we were not able to detect this in the vermis. In addition, complex spike (CS) activity showed a higher level of reciprocity with respect to SS activity in the lateral cerebellum. Although the PC activity modulated bidirectionally in both the cerebellar regions, the PCs in the lateral cerebellum that facilitated their activity, the so-called upbound PCs (De Zeeuw, 2021), contributed most prominently to the instruction of antisaccade trials. Our data add to the growing body of evidence for the role of the cerebellum in executive control and incite the field to explore the cerebellar activity under conditions where response inhibition is engaged.

## Materials and Methods

### Animals

All procedures complied with the National Institutes of Health (NIH) Guide for the Care and Use of Laboratory Animals (Bethesda, MD, United States) and were approved by the Institutional Animal Care and Use Committee of the Royal Netherlands Academy of Arts and Sciences (AVD8010020184587). Two adult male NHPs (Macaca mulatta), Mi and Mo, were used in this study.

### Surgical Procedures

Animals were prepared for eye movement and awake extracellular single in the cerebellum using surgical and electrophysiological techniques in a two-step procedure (Poort et al., 2012). Under general anesthesia induced with ketamine (15 mg/kg, i.m.) and maintained under intubation by ventilating with a mixture of 70% of N_2_O and 30% of O_2_, supplemented with 0.8% of isoflurane, fentanyl (0.005 mg/kg, i.v.), and midazolam (0.5 mg/kg ⋅ h, i.v.), we first implanted a titanium head holder to painlessly immobilize the head of the NHP. Four months later, once the NHPs had mastered the behavioral task (refer to the section below), a custom-made 40 mm chamber was implanted under the same anesthesia conditions as described above to gain access to the cerebellum with a 25° angle (Figure 1B). Animals recovered for at least 21 days before training was resumed.

### Behavioral Task

Animals were trained to perform a randomized interleaving pro- and anti-saccade task in 8 different directions (cardinal and diagonal directions), with amplitudes between 5° and 14° (Figure 1A). All recordings were conducted in complete darkness. During training and experiments, NHPs were seated in a primate chair (Crist Instrument, United States) with their head restrained at 100 cm from a screen with a resolution of 1,024 × 768 pixels. Visual stimuli were presented by a CRT-Projector Marquee 9500 LC (VDC Display Systems, Cocoa, FL, United States) with a refresh rate of 100 Hz. Binocular vision was unrestricted. A trial started when the animal fixated on a red or green fixation point at the center of the screen for a random time between 300 and 500 ms (in the figures, the colors of the fixation point and the target have been changed to blue and magenta to accommodate color blind readers). Next, a red target appeared in one of eight different target locations. After 100 ms, the fixation point changed to gray and the NHP had to perform within 500 ms either as a prosaccade toward the target (when the fixation point was red) or as an antisaccade in the opposite direction with the same amplitude (when the fixation point was green). This task design temporally separates the initial neural representation of the stimulus (300–500 ms long instruction period) from the subsequent movement execution (saccade period) (Amador et al., 1998). The animals received a liquid reward if they performed a correct saccade [within 6° of the (anti-)target] and maintained fixation for 100 ms. Within a single block, the 8 (targets) * 2 (pro/anti) possible configurations were presented in random order.

### Behavioral and Electrophysiological Recordings Setup

The position of the right eye was recorded with an infrared video-based eye tracker (iViewX Hi-Speed Primate, SMI GmbH, Teltow, Germany) at a sampling rate of 350 Hz. The eye tracker was calibrated before every recording session by having the animal look at a 1° target grid consisting of nine points (one at the center of the screen and 8 points 10° apart) to adjust the offset of *x* and *y* positions by hand. Pre- and postsurgical MRI images were used to build a 3D model of the skull and the cerebellum for the anatomical localization of the cerebellar OMV (lobules VI and VII) and the lateral cerebellum (crus I/II; Figure 1B, Sendhilnathan et al., 2020). Single-unit recordings were obtained using glass-coated tungsten electrodes (1–2 MΩ, Alpha Omega Engineering, Nazareth, Israel) through a 23-gauge guide tube, which was inserted only through the dura. A motorized microdriver (Alpha Omega Engineering, Nazareth, Israel) with a 1-mm spaced grid was used to introduce the electrode and map the recording sites with a maximum resolution of 0.25 mm. The discovery of a saccade-related site was guided by the modulating multi-unit signals during saccades. Online discrimination was done with the use of a Multi Spike Detector (MSD, Alpha Omega Engineering).

### Data Analysis Software

All analyses were performed offline using custom scripts written in MATLAB (Mathworks, Natick, MA, United States).

### Eye Movement Analysis

The eye position was sampled at 350 Hz with an infrared video eye tracker which tracked the center of mass of the pupil. The noise was reduced using a finite impulse response filter and a Savitzky–Golay filter (20 ms window). Eye speed and acceleration traces were created by differentiating the signal. Eye acceleration was further processed using a median filter. Saccade onset and offset were detected using an adaptive threshold based on 6 SD of the noise during fixation from the recording session of that day as described by Nyström and Holmqvist (2010). We removed trials with reaction times shorter than 100 ms following the “go-cue” to avoid contamination with the data from “anticipatory” saccades; these reflect a visual, involuntary reflex toward a novel stimulus in the environment and do not represent correctly planned saccades (Goldring and Fischer, 1997; Coe and Munoz, 2017).

### Electrophysiological Analysis and Statistics: Simple Spikes

Neuronal activity was identified as a single unit PC by the presence of a short pause in SS firing following a CS during at least the first 2 min of recordings (i.e., climbing fiber pause; Kitazawa et al., 1998; Catz et al., 2008; De Zeeuw et al., 2011). If the stability of the CS activity was affected later in the recordings, thereby preventing a proper identification of the climbing fiber pause, the SS activity was analyzed separately. Since we did not detect significant differences in the SS activity of the PCs with a continuous clean CS pause vs. PC recordings where CS activity was not sufficiently stable over the duration of the whole recording, all the cells have been included in the analysis of the SSs. To perform statistical analysis, the neurons were required to have at least five recorded trials per direction as well as per pro- or anti-saccadic condition. Only neurons with significant saccade-related SS activity and correct trials were incorporated in the analyses. The latter choice was based on the fact that both NHPs, the Mo and Mi, exhibited an expert level performance reaching > 90% correct responses, rendering insufficient statistical power to analyze the incorrect trials. A neuron was considered saccade-related when SS firing during the baseline period (defined as the window 400 to 50 ms before a trial onset in the intertrial interval) was significantly different from the saccade execution period activity (defined as the 150 ms time-window after the saccade onset) for at least one of the eight directions (Wilcoxon rank test; *p* < 0.05). We then computed the instantaneous SS firing rate of the neurons using a continuous spike density function (SDF) generated by convolving the spike train with a Gaussian function of σ = 50 ms width and averaging all the individual SDFs (Sendhilnathan et al., 2020). We compared the number of spikes for all pro- and anti-saccade trials of each PC using a Kolmogorov–Smirnov test and subsequently determined whether the activity of a PC was significantly different between a pro- and anti-saccade with a *p*-value < 0.05. The characteristics of the saccade-related activity, as previously reported in PC recordings, were quite heterogeneous. Based on this, we categorized the PC activity as *facilitation*, if the activity increased after the instruction or saccade onset, or as *suppression*, if the activity decreased. For the instruction period, we compared the mean firing rate of each cell from a window of 400 ms before the instruction onset to a period of 300 ms at the end of the instruction period. For the saccade period, we compared the mean firing rate of each cell from a window of 150 ms before the saccade onset to a window from the saccade onset to 150 ms after the saccade onset (Wilcoxon signed-rank, *p* < 0.05).

To determine the correlation between kinematic parameters and neuronal activity, we determined Pearson’s correlation coefficient (*r*) for all neurons in the two tasks. Modulation ratios for both areas were obtained by computing the ratio between the response during pro- and anti-saccades.

Changes in the firing rate during the instruction period were computed by taking the differences between the mean firing rates during the baseline period separately for pro- and anti-saccade trials. For the saccade period, we compared the mean activity 150 ms before the saccade onset to a period 150 ms after the saccade onset.

We performed a demixed principal component analysis (dPCA) as described by Kobak et al. (2016) to decompose the population activity into individual components and extract the dependence of the PCs on the two stimulus conditions. The dPCA data analysis tool code is available at http://github.com/machenslab/dPCA (Kobak et al., 2016). Furthermore, all standard settings were used; data were aligned to instruction offset and binned in 100 ms bins. For determining cross-validated classification accuracies, 100 iterations were used.

### Electrophysiological Analysis and Statistics: Complex Spikes

Cells were included in the analysis for complex spikes (CSs) if the firing rate of the CSs was at least 0.5 Hz over the entire recording session and if the CSs were stable for at least five trials for each of the 8 pro- and anti-saccade directions. To determine if the CS modulated to one of the task epochs, we determined whether the CS rate significantly exceeded the baseline levels during either the instruction or the saccade window (50—350 ms after the onset of the instruction and –150 to +150 ms around the onset of the saccade). The baseline firing was calculated for a period of 500 ms of inter-trial activity, and the modulation was considered significant if the firing rate exceeded ± 3 SD from the baseline.

The reciprocity of modulation between SS and CS rates was determined by finding the peak of the modulation of CS and SS responses during the same 300 ms windows (50—350 ms after the instruction onset or –150 — 150 ms around the saccade). We first calculated the peak firing rate changes for the SS and CS from the baseline (ΔCS, ΔSS) and next determined the SS–CS interaction, i.e., reciprocity (ΔCS × ΔSS). Subsequently, we used a linear regression model to find out whether there was an association between the reciprocity in the instruction period and the reciprocity during the saccade period. If extreme values exceeded three times the mean Cook’s distance, they were considered outliers and were excluded from the analysis.

## Results

### Pro- and Anti-saccades and Related Purkinje Cell Activity in the Medial and Lateral Cerebellum

We trained two adults, male rhesus macaques (referred to as NHPs, Mo and Mi) to perform randomized, interleaved pro- and anti-saccade tasks. In the prosaccade trials, the NHPs performed a (pro) saccade to a single visual target in one out of eight different locations separated 45° from each other. On the contrary, in antisaccade trials, the NHPs were trained to suppress the prepotent saccade to a visible target and perform a saccadic eye movement in the opposite direction to an unmarked, mirror position (Figure 1A, refer to section “Materials and Methods”).

A trial started with the appearance of the central fixation point (instruction period), the color of which determined the trial condition. The colors red and green were the instruction cues for the prosaccade and antisaccade movements, respectively (these colors are presented as magenta and blue in the figures to accommodate color blind readers). Following the instruction period (300–500 ms), a target appeared randomly at one of the eight different locations, while NHPs maintained fixation at the central point. After 100 ms, the central fixation point turned gray, serving as the “go-cue.” Animals had 500 ms to execute the pro- or anti-saccade eye movement (saccade period) before the trial was aborted.

Following the training period, we performed single-unit electrophysiological recordings. We recorded randomly from 90 PCs in the medial and 72 PCs in the lateral (crus-I/II) cerebellum during 122 behavioral sessions (Mi: *n* = 82 PCs [medial 28, lateral 54]; Mo: *n* = 80 PCs [medial 62, lateral 18], Figure 1B). We only considered correct trials for further analysis, as we did not have enough power to analyze error trials due to the high performance of both NHPs (94.1 ± 5.6% and 94.4 ± 5.3% for Mo and Mi, respectively). Eye movements during antisaccades exhibited longer reaction times, lower peak velocities, larger amplitudes, and longer durations than prosaccades (Figure 1C).

A neuron was considered saccade-related if the SS activity during the intertrial interval (baseline period) was significantly different from that during the saccade execution period (i.e., from the saccade onset to 150 ms after the saccade onset) for at least one of the eight directions (refer to section “Materials and Methods”). Approximately, half of the cells recorded in both areas fulfilled this criterion (*n* = 40 [44%] for the medial, and *n* = 38 [53%] for the lateral cerebellum). All subsequent analyses were performed only for these cells unless noted otherwise (*n* = 78 PCs). The average number of trials per cell in the medial cerebellum was 75 ± 49 SD and 71 ± 47 SD for pro- and anti-saccades, respectively, while in the lateral cerebellum, these numbers were 80 ± 52 SD and 76 ± 50 SD, respectively.

To assess whether there was a correlation between saccade parameters and PC SS firing rate, we regressed the firing rates for each cell in the population (i.e., 90 PCs in the medial and 72 PCs in the lateral cerebellum) in the two recording areas to amplitude or peak speed and compared the corrected firing rate using the regression coefficient (ß) between pro- and anti-saccade (corrected firing = firing rate/regression coefficient). We found no significant correlations between the SS firing rate and saccade amplitude or saccade peak speed in neither the medial nor the lateral cerebellum (Figure 1D). This allowed us to directly compare pro- and anti-saccade trials between the two areas by pooling the mean firing rates of each cell in each condition. The SS activity during the saccade execution was significantly higher in the lateral cerebellum compared to that in the medial cerebellum for both pro- and anti-saccade trials (mean and SD, medial pro 66.7 ± 30 spks/s vs. lateral pro 69.3 ± 39 spks/s, *p* = 0.04; medial anti 68 ± 36 spks/s vs. lateral anti 71.8 ± 39 spks/s, *p* = 0.03; *n* = 90 *M*edial, *n* = 72 lateral, Wilcoxon rank-sum test) (Figure 1E). Further, Cohen’s *d* suggests a moderate significance (medial pro vs. lateral pro = 0.405, medial anti vs. lateral anti = 0.331).

### Purkinje Cells in Both the Medial and Lateral Cerebellum Modulate Their Activity During the Task but With Different Characteristics

**FIGURE 2:**
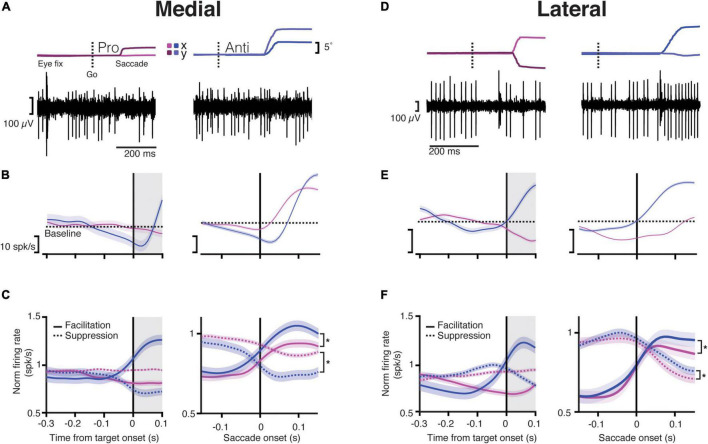
Purkinje cells in both the medial and lateral cerebellum start modulating their activity during the instruction period. **(A)** Top, Eye trace for an example trial during the saccade onset for a pro- (left) and anti-saccade (right). The dashed line indicates the time of the “go” cue. Dark and light magenta and blue show the *x* and *y* positions (horizontal and vertical components) of the eye trace for pro- and anti-saccades, respectively; bottom, traces of activity recorded from a PC in the medial cerebellum for a single trial aligned to the eye trace indicated above. Note that the traces include many simple spikes (SSs; smaller spikes) and only a few complex spikes (CSs, bigger spikes). **(B)** Top, Raster plots of trials showing the activity of five different neurons aligned to the end of the instruction period (target onset; left) or to the saccade onset (right) for prosaccades (magenta) and antisaccades (blue) for the medial cerebellum; bottom, the average response time-course for one example neuron aligned to the same periods during the same conditions. The dashed line shows the baseline mean. Shaded regions denote SEM. Gray shaded areas show the time after the instruction period when the target appears, but the animals must keep fixating on the central dot. **(C)** Time-course of normalized responses averaged for all neurons in the medial cerebellum that either increased their firing (*facilitation* cells, solid line) or decreased it (*suppression* cells, dashed lines) aligned with the target onset (left) or the saccade onset (right). Asterisk denotes significant statistical comparisons after the saccade onset, facilitation pro vs. anti: *p* = 1.61 × 10^–7^, suppression pro vs. anti: *p* = 5.3 × 10^–23^. **(D)** Same as in **(A)** but for lateral cerebellum. **(E)** Same as in **(B)** but for lateral cerebellum. **(F)** Same as in **(C)** for the lateral cerebellum (asterisk denotes significant difference between facilitation pro vs. anti: *p* = 1.08 × 10^–17^, suppression pro vs. anti: *p* = 4.4 × 10^–9^).

The activity profile of the PCs followed roughly one of the two patterns; they either increased or decreased their activity following the saccade onset. Accordingly, we classified these cells as *facilitation* or *suppression* PCs, respectively (Figure 2). The activity profile of the PCs was the same during pro- and anti-saccades in both areas, meaning that if one PC increased its firing rate after the saccade execution for prosaccades, it also increased the firing rate for antisaccades. This was true for all PCs except for two in the lateral cerebellum, which showed a mixed firing behavior; these two cells were included in their respective category for further analyses (e.g., as a *facilitation* cell in prosaccade trials and a *suppression* cell in antisaccade trials).

We next assessed the SS activity during the instruction period (for details, refer to section “Materials and Methods”). Given the variable duration of the instruction period, which ranged between 300 and 500 ms, we analyzed the last 300 ms of this epoch, before the target onset (Figure 1A). Here, we also encountered diverse neural responses in both regions. We classified the activity of these neurons in the same way as for the saccade period, depending on whether they increased (*facilitation* PCs), or decreased (*suppression* PCs) their SS firing during the instruction period compared to the activity during the intertrial interval. Compared to the lateral cerebellum, the medial cerebellum had overall less *facilitation* PCs (medial pro 10%, medial anti ∼11%, lateral pro ∼17%, lateral anti ∼18%) and *suppression* PCs (medial pro ∼11%, medial anti ∼7%, lateral pro ∼14%, lateral anti ∼15%) during the instruction of pro- and anti-saccade trials (Figures 2B–F). The remaining cells had no change in the firing rates during the instruction period (medial: ∼61%, lateral 36%).

We next looked at the timing of the SS activity of *facilitation* and *suppression* PCs between the medial and lateral cerebellum in relation to the saccade onset, pooling the pro- and anti-saccade conditions (Figure 2). Latencies were defined as the time between the saccade onset and the maximum (peak) or minimum (trough) in the firing rate presented after this onset. We found that the latencies of the *suppression* PCs in the lateral cerebellum, measured at the trough after the saccade onset, were significantly longer than those in the medial cerebellum (mean ± SD lateral 133 ± 40 ms vs. medial 89 ± 68 ms, *p* = 0.01; lateral *n* = 38, medial *n* = 40, Wilcoxon rank-sum, Figures 2C,F). In contrast, the *facilitation* PCs showed similar timing of the peak activity in the medial and the lateral cerebellum (mean ± SD medial 105 ± 41 ms vs. lateral pro 84 ± 48 ms; *p* = 0.4; Wilcoxon rank-sum). There were no differences between prosaccades and antisaccades in this respect, neither for *suppression* PCs (mean ± SD medial pro 87 ± 70 ms vs. medial anti 93 ± 67 ms, *p* = 0.83; lateral pro 139 ± 40 ms vs. lateral anti 127 ± 42 s; *p* = 0.1; Wilcoxon rank-sum test) nor for *facilitation* PCs (mean ± SD medial pro 105 ± 41 ms vs. medial anti 104 ± 33 ms, *p* = 0.2; lateral pro 84 ± 48 ms vs. lateral anti 106 ± 43 ms, *p* = 0.9; Wilcoxon rank-sum test).

Together, these results suggest that PCs in the lateral cerebellum are more prominently involved in the instruction of planned saccades than those in the medial cerebellum and that the course of SS responses of *suppression* PCs in the lateral cerebellum during the subsequent saccade period is delayed with respect to that in the medial cerebellum.

### Purkinje Cells in the Lateral Cerebellum Show a Stronger Modulation During the Instruction Period

**FIGURE 3:**
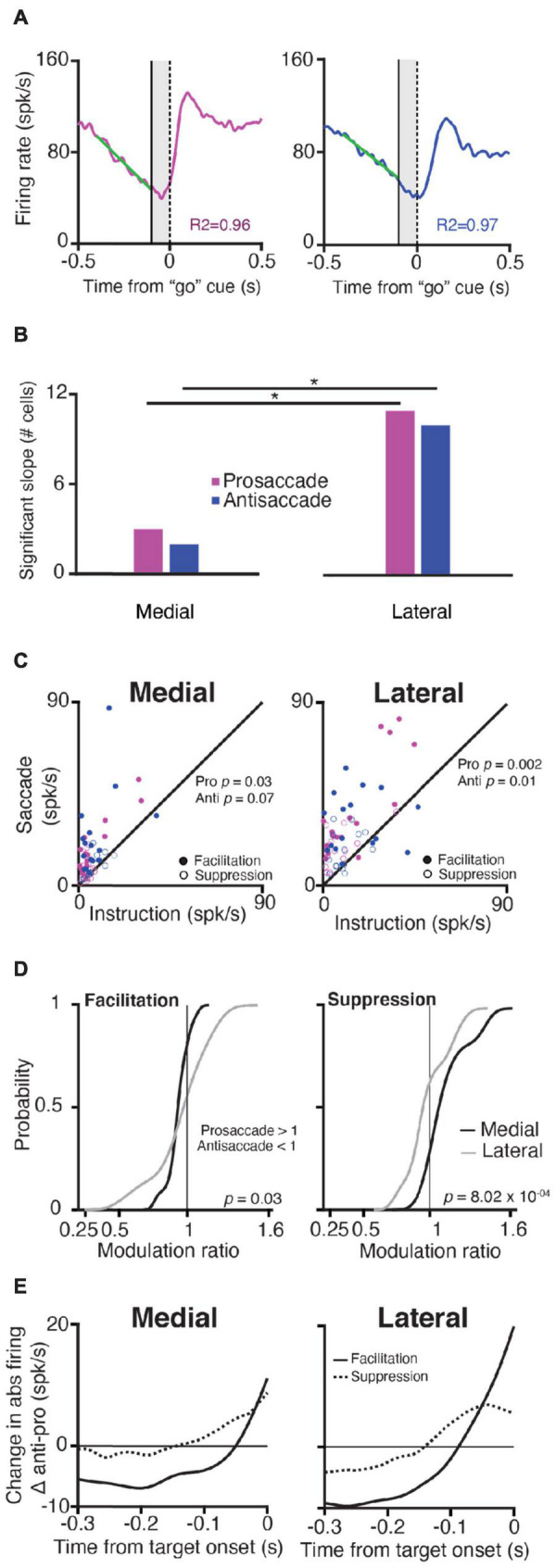
Purkinje cells in the lateral cerebellum exhibit stronger modulation than PCs in the medial cerebellum during the instruction period. **(A)** Explanation of slope calculations used for panel **(B)**; *left*, Time-course of SS responses during prosaccades (magenta) and antisaccades (blue); for example, a neuron in the lateral cerebellum aligned to the “go” cue (vertical dashed line at zero); right, *the* activity of the same neuron, but now for an antisaccade trial aligned to the “go” cue. A solid vertical line shows the end of the instruction period, the shaded area denotes the appearance of the target but the NHPs must keep fixating. A linear regression model was fitted (green diagonal solid lines) to the firing rate of 300 ms before the end of the instruction period to calculate the slopes (refer to section “Materials and Methods”). **(B)** Number of cells with significant slope modulation in the medial and lateral cerebellum (χ2 test for proportions) during pro- and anti-saccades (*p* = 0.02 for both comparisons). **(C)** Scatter plots of maximum SS firing rate of *facilitation* and *suppression* PCs during the instruction period vs. that during the saccade period (in spks/s) for pro- and anti-saccades in the medial and lateral cerebellum. **(D)** Cumulative distribution of the SS modulation ratio for the medial and lateral cerebellum for *facilitation* (left) and *suppressive* (right) PCs. Insets show *p*-values for the comparison between the medial and lateral cerebellum (two-sample K-S test). **(E)** Difference in change in the absolute firing rate between pro- and anti-saccades (abs antisaccade SS activity and abs prosaccade SS activity) for *facilitation* and *suppression* neurons in the medial and lateral cerebellum from the target onset.

We observed that in some PCs, the SS modulation ramped throughout the interval between the instruction and the saccade epoch (Figures 2B–E, 3A). We next tested if these saccade-related cells also modulated their activity during the instruction period. For this reason, we quantified the ramping of SS activity by fitting a linear model to the firing rate during the last 300 ms of the instruction period, and we classified PCs as ramping cells when *R*^2^ was higher than 0.75. We found that the ramping modulation was quite rare in the medial cerebellum and more prevalent in the lateral cerebellum (Figure 3B); this was held true for both the prosaccade and antisaccade trials [medial *n* (pro) = 3, *n* (anti) = 2; lateral *n* (pro) = 11, *n* (anti) = 10; both comparisons *p* = 0.02; χ^2^ test]. Overall, we found more PCs that significantly changed their firing from the baseline during the instruction period in the lateral cerebellum than in the medial cerebellum (medial vs. lateral, *p* = 0.03; Mann–Whitney test), suggesting a more prominent role for PC modulation in the lateral cerebellum during instruction.

When we investigated the changes in the firing rate (modulation, see section “Materials and Methods”) for each PC during the instruction and saccade period within the medial and lateral cerebellum, we found that in the lateral cerebellum, the maximum change in the firing rate during the instruction period was significantly different from that during the saccade period, for both pro- and anti-saccade trials (Lateral pro *p* = 0.002, anti *p* = 0.01; Wilcoxon rank-sum test) (Figure 3C). In the medial cerebellum, only the prosaccade trials showed this trend (Medial pro *p* = 0.03, anti *p* = 0.07, Wilcoxon rank-sum test) (Figure 3C).

Further examination of the differences in responses during pro- and anti-saccades showed that *facilitation* and *suppression* cells in the lateral cerebellum show the significant encoding of SS modulation for trial conditions. We calculated a modulation ratio between the responses during the saccade period in the two types of trials, defined as the ratio between the response during pro- and anti-saccades (Figure 3D, see section “Materials and Methods”). Values of 1 indicate that both the responses are the same, values higher than 1 indicate that the prosaccade activity was higher, and below 1 indicates that antisaccade activity was higher. Medial PCs had somewhat different responses when comparing pro- and anti-saccade activity in contrast to lateral PCs (mean of *facilitation* PCs in the medial cerebellum 0.91 ± 0.1 SD vs. that in the lateral cerebellum 0.88 ± 0.3, *p* = 0.03; mean of *suppression* PCs in the medial cerebellum 1.16 ± 0.2 vs. that in lateral cerebellum 1 ± 0.2, *p* = 8.02 × 10^–04^; one-tailed K-S test). To summarize, PCs in both the medial and lateral cerebellum modulate their activity during the instruction of pro- and anti-saccades, with a relatively bigger role for the *facilitation* cells during the instruction of antisaccades. Yet, PCs in the lateral cerebellum differ from those in the medial cerebellum in that they have a stronger modulation during both the instruction and saccade periods and for the pro- vs. anti-saccade conditions.

### Differences Between Facilitation and Suppression Cells That Occur in Both the Medial and Lateral Cerebellum

In addition to the observed differences across the recorded regions, we identified features that were shared across the medial and lateral cerebellum. First, we observed that *facilitation* and *suppression* PCs in the medial and the lateral cerebellum operate at different baseline frequencies, which is in line with the upbound and downbound microzones of PCs described for the cerebellar cortex of rodents (De Zeeuw, 2021). Since these microzones have been hypothesized to operate at a relatively low and high baseline of SS firing frequencies, respectively, so as to leave ample room for increases (i.e., an upbound effect at the level of the PCs) and decreases (i.e., a downbound effect at the level of the PCs) during epochs of stimulation and modulation, we investigated whether the *suppression* and *facilitation* cells differed in this respect (Sun et al., 2017; Herzfeld et al., 2018; Soetedjo et al., 2019). We found that *suppression* cells started at a relatively high baseline SS firing frequency in both the medial and lateral cerebellum (62.43 ± 3.76 spks/s and 69.6 ± 6.12 spks/s, respectively), and *facilitation* cells started at a significantly lower baseline SS firing frequency in both the regions (56.36 ± 3.65 spks/s and 54.72 ± 3.76 spks/s, *p* = 0.02; one-tailed Wilcoxon rank-sum test comparison between all *facilitation* vs. *suppression* cells pooled in both areas), in line with the concept of upbound and downbound microzones (De Zeeuw, 2021).

Next, we found that whereas the saccade period contained more *suppression* PCs, during the instruction period, the contribution of the *facilitation* cells appeared most prominent, particularly during that of the antisaccade trials. When we subtracted the SS modulation during the prosaccade trails from the activity during the antisaccade trials (antisaccade SS activity and prosaccade SS activity), *facilitation* PCs in both the medial and lateral cerebellum showed a larger change in the firing rate just before the target onset during the instruction period (Figure 3E). These changes were significantly less pronounced for *suppression* cells (*facilitation* PCs vs. *suppression* PCs in the medial cerebellum *p* = 4.4 × 10-6; *facilitation* PCs vs. *suppression* PCs in the lateral cerebellum *p* = 0.004; Wilcoxon rank-sum test). Thus, *facilitation* and *suppression* cells can be characterized by several features that are common for the medial and lateral cerebellum and their existence suggests a PC mechanism to exert the inhibition or disinhibition of the cerebellar nuclei neurons for the generation of saccades (Ishikawa et al., 2014), in line with the concept of upbound and downbound microcomplexes, which encompass both the inhibitory PCs and their target neurons in the cerebellar nuclei (De Zeeuw, 2021).

### The Lateral but Not the Medial Cerebellum Contains Information About the Stimulus Identity During the Instruction Period

To extract the features of the population activity related to pro- and anti-saccades, we applied a linear dimensionality reduction technique: (dPCA; Kobak et al., 2016). The dPCA demixes the neural activity into activity related to the task components that capture the most variance in the data. The dPCA was used to investigate to what extent the neurons are tuned to two stimulus conditions indicated by the color of the fixation point (i.e., the pro- or anti-saccades), or whether their activity is condition-independent. In the condition-independent group, dPCA components show task-related activity modulations that cannot be assigned to one of the stimulus conditions. As explained above, due to the high behavioral performance of both NHPs, our dataset lacked the power to create another category with decision-related components in the correct/incorrect trials. The dPCA was applied to the entire population of recorded cells in the medial (*n* = 90) and the lateral cerebellum (*n* = 72). We included cells that had at least 5 trials in all pro- and anti-saccade conditions. The results were cross-validated to measure time-dependent classification accuracy, and a shuffling procedure was applied to assess whether the classification accuracy was significantly above the chance level (refer to section “Materials and Methods”).

**FIGURE 4:**
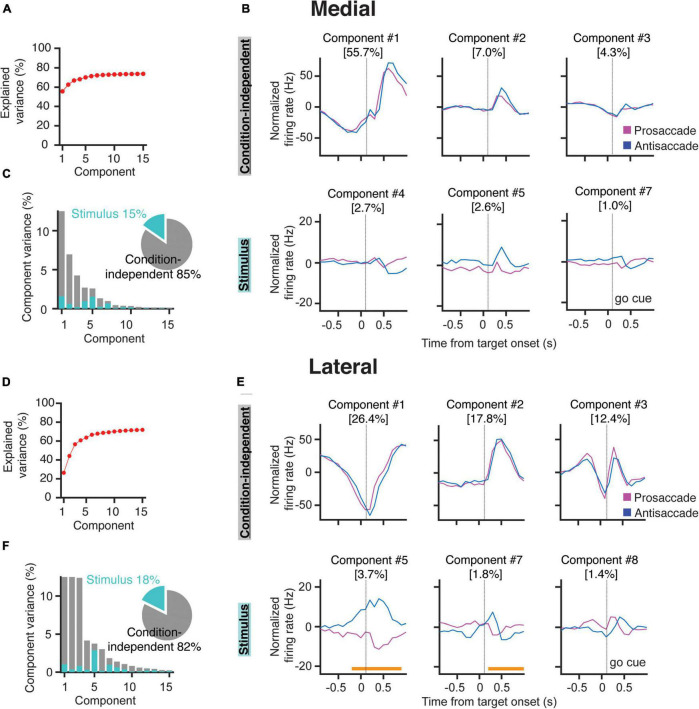
Demixed PCA indicates that PCs in the lateral cerebellum encode stimulus identity. **(A)** Cumulative fraction of variance explained by dPCA in the medial cerebellum. The dotted line indicates an estimate of the fraction of variance in the data explained by the dPCA. **(B)** Projections of the PSTH’s of all the medial cerebellar PCs onto the most prominent decoding axes; top, first three condition-independent components; bottom, first three stimulus-related (pro/anti) components. Percentages denote the variance captured by each component. Time from instruction offset. **(C)** Variance of the individual dPCAs for the medial cerebellum. Each bar shows the proportion of the total variance. Gray bars depict condition-independent variance and cyan bars indicate stimulus-related (pro/anti) variance. The pie chart shows the total signal variance for stimulus-related and condition-independent components. **(D–F)** Same as in **(A–C)** but for the lateral cerebellum, orange horizontal bars in **(E)** indicate the time during which the stimulus can be decoded from the population firing rate.

In the medial cerebellum, the overall variance explained by the dPCA components was mostly captured by the first three components, which were independent of the stimulus condition, and together they explained 66.9% of the variance (Figure 4A). These components all have strong activity after the end of the instruction, and thus around the time, the animal initiates the saccade (Figure 4B). Components 4, 5, and 7 were selected as containing the strongest activity differences in relation to the stimulus conditions (Figure 4B, bottom row and Figure 4C). To determine the significance, each component was used as a linear decoder to classify each of the stimulus conditions (refer to section “Materials and Methods”). No components could be reliably used to classify the stimulus condition in the medial cerebellum.

Similar to the medial cerebellum, components 1, 2, and 3 of the lateral cerebellum captured most of the variance in the condition-independent categories (56.4%, Figures 4D,E). These components also showed a strong modulation after the instruction offset. In contrast to the medial cerebellum, two of these components are modulated during most of the instruction period (Figure 4F). Stimulus-related components showed significant tuning for pro- and/or anti-saccades in components 5 and 7. Notably, in component 5, a stimulus condition classification was already possible, almost 200 ms before the end of the instruction period. In the lateral cerebellum, the variance in this component could almost be entirely attributed to the stimulus conditions (Figure 4F). Indeed, in component 5, the time period in which the stimulus condition could be decoded from the firing rates was broad, bridging the instruction period to the saccade. This appears to be in line with the prominent ramping modulation described in Figure 3. These results show that PCs in the lateral, but not in the medial, cerebellum contain reliable information about the stimulus identity during the instruction period.

### Complex Spike Responses of Purkinje Cells in the Lateral Cerebellum Show Reciprocal Activity With Simple Spike Responses

**FIGURE 5:**
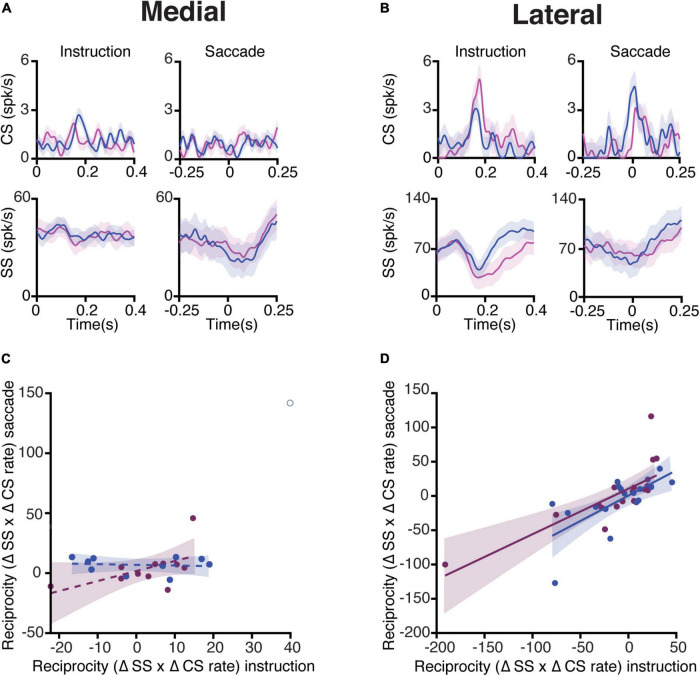
Complex spike and simple spike interaction during pro- and anti-saccade trials. **(A)** Top, average CS rate of PCs in the medial cerebellum during prosaccades (magenta) and antisaccades (blue) aligned to the onset of the instruction (left) and saccade (right) periods. The bottom panels show the associated SS responses. Shaded areas represent the standard error of the mean (SEM). **(B)** Same as **(A)**, but for neurons recorded in the lateral cerebellum. **(C)** Relation of reciprocity between CS and SS responses during the instruction and saccade periods. To quantify the reciprocity, we calculated the maximum change in the CS firing rate in the windows of interest (instruction and saccade periods), the associated maximum change in the SS rate in a window of 300 ms around the peak of CS activity, and subsequently the product of the two (Δ CS rate × Δ SS rate). Note that if a positive modulation of CSs is associated with a negative modulation of SSs, the reciprocity measure presented here will give a negative value. If CSs and SSs show simultaneous positive modulations, and thus no reciprocity, this will result in a positive value. Every dot represents one cell. The coefficients of determination (*R*^2^) are as follows: medial pro = 0.03, medial anti = 0.01. **(D)** Same as **(C)** but of lateral neurons, coefficients of determination: lateral pro = 0.54, and lateral anti = 0.42. Shading indicates a 95% CI of the regression lines.

In a subset of recorded PCs, we were able to reliably isolate and analyze CS responses throughout all trials. We restricted the CS analysis only to cells with average CS firing rates higher than 0.5 Hz over the duration of the whole recording (medial *n* = 11, lateral *n* = 18). Similar to the SS analyses, we examined the CS responses separately in the medial and lateral cerebellum during the instruction and saccade periods (Figure 5). The average CS activities during the instruction (1.10 Hz ± 0.47 SD, *n* = 11) and saccade (1.11 Hz ± 0.50 SD, *n* = 11) periods in the medial cerebellum were not significantly different from those in the lateral cerebellum (1.15 Hz ± 0.33 SD, *n* = 18 and 1.13 Hz ± 0.52 SD, *n* = 18, respectively; *p* = 0.87 and *p* = 0.36 for the comparison of medial vs. lateral during the instruction and saccade periods, respectively; Wilcoxon rank-sum).

Next, we quantified the interaction between CS and SS responses during the instruction period (Figures 5A,B, left panels). For every PC of both the medial and lateral cerebellum, we calculated the maximum change in the CS firing rate from the baseline in the instruction window and the maximum change in the SS firing rate from the baseline in the 300 ms window around the time of the peak CS rate. This was also done for the saccade period for the pro- and anti-saccade trials separately. A change in the CS firing from the baseline was multiplied by the change in the SS firing from the baseline to establish their interdependence, i.e., reciprocity (Badura et al., 2013). To determine whether reciprocity was dependent on both epochs, a linear regression was applied to the reciprocity values in the instruction and saccade windows (Figures 5C,D). Lateral cerebellar PCs had a positive association with reciprocity during both the instruction and the saccade periods, whereas the medial cerebellum did not (coefficients of determination (*R*^2^): lateral pro 0.54 (*n* = 16), and lateral anti 0.42 (*n* = 16), medial pro 0.03 (*n* = 10), and medial anti 0.01 (*n* = 9). Due to the relative lack of error trials, unfortunately, we were not able to analyze the error-triggered CSs with sufficient power in the direction of the selective error context, as has been successfully done by others (Herzfeld et al., 2018).

## Discussion

The antisaccade task has been used to investigate flexible behavioral control, where a correct suppression of a prosaccade followed by a response to the opposite direction forms a feature of executive control (Mokler and Fischer, 1999; Mitchell et al., 2002). We found evidence for the cerebellar involvement in the volitional control of this behavior in both the medial (i.e., OMV) and lateral (i.e., crus I/II) cerebellar cortex by recording the activity of PCs during pro- and anti-saccades in NHPs. Our findings add to the growing body of evidence pointing to an important contribution of the cerebellum to cognitive processes in general and to voluntary motor behavior in particular. We demonstrated that PCs in both areas modulate their activity depending on the trial type and that SS responses of populations of PCs in the lateral cerebellum are able to discriminate the type of trial before the eye movement takes place. Even though the lateral cerebellum is not traditionally viewed as a saccade-control area, our findings support early reports where saccades are elicited following the electrical stimulation of the lateral regions of the hemispheres, especially in crus I/II (Ron and Robinson, 1973).

Antisaccades exhibit kinematic properties that differ from prosaccades, a result consistent with previous reports on humans and NHPs (Amador et al., 1998; Munoz and Everling, 2004). Even so, the preferential modulations of SS responses in the PCs were not correlated with any of those kinematic changes. This suggests that the observed activity was not merely an efference copy of the motor command but reflects the preparatory or planning phase of the voluntary movement in which the PC firing is determined by the cue. This is in line with the recent findings in patients with spinocerebellar ataxia type 2 and the late onset of cerebellar ataxia, who are presented with deficits in executive functions during an antisaccade test (Pretegiani et al., 2018) and in patients with cerebellar atrophy who show impairments when making goal-oriented movements (Piu et al., 2019). To what extent the SS responses in our study also correlate with the presence or absence of reward at the end of the trials remains to be seen. Due to the low number of error trials, it is impossible to analyze the difference between the correct and incorrect trials in a statistically meaningful way. It will be interesting to focus on this possibility in the future in less well-trained NHPs, so as to be able to compare the CS and SS reward signaling in NHPs vs. rodents (Heffley and Hull, 2019).

Various parameters of PC activity differed between the medial and lateral cerebellum. The SS activity of PCs in the lateral cerebellum (1) displayed more signal separation/selectivity for pro- and anti-saccades during the instruction period, suggesting that the population contains information about the stimulus identity, and (2) showed a greater firing rate during the execution of saccades. Likewise, their CS activity showed a higher level of reciprocity with respect to SS activity, during both the instruction and execution periods. Together, these data indicate that different modules of different cerebellar regions can contribute to the same complex behavior, yet with different propensities.

### Purkinje Cell Modulation During the Instruction Period

Studies from cerebellar patients suggest that the cerebellum might not be involved in the initiation of saccades, but in the correct and accurate execution of them (Kornhuber, 1971; Thier et al., 2002). Our results indicate that PCs in the lateral cerebellum encode information about the stimulus identity. The dPCA analysis of crus I/II PCs exhibited significantly different SS activity between both conditions, showing that these contain sufficient information to distinguish a pro- from an antisaccade task. This is supported by a previous study showing that downstream neurons in the dentate nucleus modulate during the preparation of antisaccades. and that the inactivation thereof promotes antisaccade errors (Kunimatsu et al., 2016). This instruction-related activity in antisaccade trials may be relayed to brainstem nuclei that are engaged in curtailing reflexive saccades (Everling et al., 1999). The persistent SS modulation of PCs in the lateral cerebellum upon cue presentation suggests that it updates an internal model for well-timed activity during motor planning, which may be relayed to the prefrontal cortex (Gao et al., 2018).

Correct decoding of a visual stimulus is an essential component of the pro- and anti-saccade tasks. Visual responses in the cerebellum have been reported in several areas including the floccular complex, crus I/II, and the inferior semilunar lobe (VII/VIII; Marple-Horvat and Stein, 1990). Dentate nucleus neurons have an activity bridging the period from task instructions to motor execution (Kunimatsu et al., 2016), which could be derived from the same population of PCs as we recorded in the lateral cerebellum. Our results on the CS-SS reciprocity in the lateral cerebellum raise the possibility that CS responses play a similar role in processing visual stimuli for the preparation of ensuing movements. Processing of both visual stimuli and motor functions in the same cells could reduce reaction times, and thus lead to more efficient behavior.

Contemporary models of antisaccade decision-making require evidence accumulation to reach a decision boundary in the frontal cortex (Munoz and Everling, 2004). PCs receive diverse sensory, motor, and cognitive information through their parallel fiber inputs, which may facilitate efficient evidence accumulation. Classical models of cerebellar function indicate that climbing fiber-mediated plasticity in the molecular layer determines which parallel fiber inputs are turned into SS modulation (Gao et al., 2012). The PCs that we measured in the lateral cerebellum carry both SS and CS signals during the instruction period of the antisaccade task. We hypothesize that through the pre-learned associations of parallel fiber and climbing fiber signals, relevant evidence from the stimulus (i.e., dot color in instruction) for the selection of the required action (i.e., pro- or anti-saccade) can be rapidly relayed from the lateral cerebellum to the frontal saccade areas. In cerebellar patients, latencies for antisaccades are prolonged, indicating a less efficient decision-making process in the absence of cerebellar input (Pretegiani et al., 2018; Piu et al., 2019).

### Purkinje Cell Modulation During Saccade Execution

Single PCs modulated their SS activity during the execution of pro- and anti-saccades in both the medial and lateral cerebellum, but only lateral PCs were able to successfully discriminate the type of trial. Neurons that facilitated or suppressed their response after saccade onset exhibited differential activity during pro- and anti-saccade trials (Figure 3D). As a population, PC responses were different when executing a saccade toward a visible target than when using an internally generated goal. This is interesting for two reasons: first, the medial cerebellum has always been identified as a motor and timing controller for the different types of saccades, interacting with areas in the cortex and the brainstem (McElligott and Keller, 1984; Noda and Fujikado, 1987; Thier and Möck, 2006; Herzfeld et al., 2015, 2018), while the role of the lateral cerebellum in the saccade control has remained under debate. Indeed, lesions of the lateral cerebellum have been shown to delay the onset of saccades between 10 and 60 ms and result in variable hypo- and hypermetria (Ohki et al., 2009), but the contribution of PC activity in the lateral cerebellum to proactive control of saccades and flexible behavioral in general had not been conclusively proven. Second, differential cerebellar activity during antisaccades implies that it contains neural signatures to modulate the preparation and execution by encoding information about the current context of the upcoming action (saccade), which is necessary for the fast classification of the response as correct or erroneous. This finding is in line with the impact of focal cerebellar lesions on humans in the changes in potentials observed during performance monitoring in an antisaccade task (Peterburs et al., 2012).

### Cerebellar Modules Operate in Parallel

The data on *suppression* PCs in the medial cerebellum showing a relatively strong SS modulation during an early execution of both pro- and anti-saccades as well as those on *facilitation* PCs in the lateral cerebellum showing a prominent modulation at the end of the instruction of antisaccades indicate that different modules of different cerebellar regions can both contribute to the same complex behavior during specific windows of the task. A similar conclusion was drawn from a recent study on delay eyeblink conditioning (Wang et al., 2020). Even though this form of conditioning is classically considered to be controlled solely by modules in the lobule simplex of the lateral cerebellum, Wang et al. (2020) have shown that modules of the medial cerebellum are equally essential, yet also contributed to a slightly differential fashion. Whereas the eyeblink module in the lateral cerebellum may only control the conditioned response (ten Brinke et al., 2015), that in the medial cerebellum may regulate mainly muscle tone, affecting not only the conditioned response but also the unconditioned response (Wang et al., 2020). Similar to the current pro- and anti-saccade tasks, during the eyeblink conditioning, the medial and lateral cerebellum can also both engage *facilitation (i.e., upbound)* and *suppression* (i.e., *downbound)* cells, and they also operate at relatively low and high baseline firing frequencies, respectively (ten Brinke et al., 2015; Wang et al., 2020). Our current finding that *facilitation* and *suppression* cells appear to play a more dominant role during the instruction and execution of the saccade task, respectively, further highlights the differential functional relevance of the upbound and downbound modules (De Zeeuw, 2021).

The current data are compatible with the possibility that modules in the medial regions of the oculomotor cerebellum contribute mainly, but not exclusively, to the fine control of saccade dynamics and endpoints (Robinson and Fuchs, 2001; Thier et al., 2002; Herzfeld et al., 2015; Soetedjo et al., 2019), while the lateral cerebellum may be more concerned with higher cognitive functions, such as preparatory processes that occur early after the stimulus onset (Chabrol et al., 2019). Indeed, the lateral regions of the cerebellum appear well connected to neocortical regions that contribute to complex forms of error detection and behavioral adjustment, and they can show activity changes that also correlate with events before the movement execution (Mano et al., 1991; Ashmore and Sommer, 2013; Kunimatsu et al., 2016).

The main target of PC microzones in the lateral cerebellum is the dentate nucleus (Chabrol et al., 2019). Similar to the neurons in the interposed nucleus that are involved in eyeblink conditioning (Ten Brinke et al., 2017), the neurons in the dentate nucleus that are involved in saccade-related behavior can show increases and decreases (Ashmore and Sommer, 2013; Kunimatsu et al., 2018). Kunimatsu et al. (2018) found only upward modulating units during self-initiated saccades, but Ashmore and Sommer (2013) revealed groups of both *facilitation* and *suppression* cells during delayed saccades. Likewise, during wrist movements, dentate neurons can show both increases and increases, albeit with a preponderance of disinhibited cells (Ishikawa et al., 2014). Thus, given the upbound and downbound activity in PCs of the lateral cerebellum found in the current study, these differences downstream in the dentate nucleus may reflect differences in the behavioral paradigms and the microcomplexes involved (De Zeeuw, 2021).

In this respect, it will be interesting to find out to what extent the *suppression* and *facilitation* of PCs can be associated with concurrent disinhibition and/or subsequent rebound excitation in the nuclei, with or without the activation of the mossy fiber and climbing fiber collaterals (De Zeeuw et al., 2011; Witter et al., 2013; Ishikawa et al., 2014). During the antisaccade test, the prevalence of *facilitation* PCs during the instruction period may provide a relatively strong suppression of cerebellar nuclei cells (fastigial or dentate nuclei), possibly suppressing the premature generation of saccades *via* direct inhibition. The dominance of *suppression* PCs during the saccade period, on the other hand, may provide the necessary release of cerebellar nuclei from the inhibition of PCs for saccade generation, possibly supported by activated mossy fiber collaterals. Further studies should evaluate the simultaneous activity of both PCs and cerebellar nuclei neurons from the different upbound and downbound microcomplexes, elucidating the main cellular mechanisms that are relevant downstream in the nuclei during specific behaviors (Ishikawa et al., 2014; Ten Brinke et al., 2017; Chabrol et al., 2019).

## Significance Statement

The antisaccade task is commonly used in research and clinical evaluation as a test of volitional and flexible control of behavior. It requires a volitional suppression of prosaccades, a function that has been attributed to the neocortex. However, recent findings indicate that the cerebellum also contributes to this behavior. We recorded neurons in the medial and the lateral cerebellum to evaluate their responses to this task. We found that both regions significantly modulated their activity during this task, but only cells in the lateral cerebellum encoded the stimulus identity in each trial. These results indicate that the cerebellum actively contributes to the control of flexible behavior and that the lateral and the medial cerebellum play different roles during volitional eye movements.

## Data Availability Statement

The raw data supporting the conclusions of this article will be made available by the authors, without undue reservation.

## Ethics Statement

The animal study was reviewed and approved by Royal Netherlands Academy of Arts and Sciences (AVD8010020184587).

## Author Contributions

CD, AB, MF, NF, and EA designed the study and analysis. EA and PR performed the surgeries on the animals. EA and NF executed the experiments. EA, NF, AB, and PH analyzed the data. EA, NF, AB, and CD wrote the first draft. All authors edited the manuscript.

## Conflict of Interest

The authors declare that the research was conducted in the absence of any commercial or financial relationships that could be construed as a potential conflict of interest.

## Publisher’s Note

All claims expressed in this article are solely those of the authors and do not necessarily represent those of their affiliated organizations, or those of the publisher, the editors and the reviewers. Any product that may be evaluated in this article, or claim that may be made by its manufacturer, is not guaranteed or endorsed by the publisher.
